# 2-Deoxyglucose, an Inhibitor of Glycolysis, Enhances the Oncolytic Effect of Coxsackievirus

**DOI:** 10.3390/cancers14225611

**Published:** 2022-11-15

**Authors:** Pavel O. Vorobyev, Dmitry V. Kochetkov, Peter M. Chumakov, Natalia F. Zakirova, Sofia I. Zotova-Nefedorova, Konstantin V. Vasilenko, Olga N. Alekseeva, Sergey N. Kochetkov, Birke Bartosch, Anastasiya V. Lipatova, Alexander V. Ivanov

**Affiliations:** 1 Center for Precision Genome Editing and Genetic Technologies for Biomedicine, Engelhardt Institute of Molecular Biology, Russian Academy of Sciences, 119991 Moscow, Russia; 2 Faculty of General Medicine, Pirogov Russian National Medical University, 117997 Moscow, Russia; 3 INSERM U1052, CNRS UMR-5286, Cancer Research Center of Lyon (CRCL), 69003 Lyon, France; 4 University of Lyon, Université Claude-Bernard (UCBL), 69001 Lyon, France

**Keywords:** oncolytic virus, coxsackievirus, metabolism, 2-deoxyglucose, seahorse

## Abstract

**Simple Summary:**

Oncolytic viruses infect tumor cells and trigger their death and elimination by the immune system. Several viral strains have been introduced into clinical practice. However, their therapeutic efficacy remains controversial. Here, we show that Coxsackievirus B5 (CVB5) causes the death of glioblastoma cells. Interestingly, 2-deoxyglucose, an inhibitor of glycolysis augments the oncolytic effect induced by CVB5. This synergism occurs in cells with a high respiratory phenotype and increased glycolytic capacity. Thus, 2-deoxyglucose or its analogs can be evaluated further as drugs to potentiate the oncolytic effects of enteroviruses against glioblastoma multiforme.

**Abstract:**

Glioblastoma multiforme (GBM) is one of the most common types of brain tumor. Despite intensive research, patients with GBM have a poor prognosis due to a very high rate of relapse and significant side effects of the treatment, with a median survival of 14.6 months. Oncolytic viruses are considered a promising strategy to eliminate GBM and other types of cancer, and several viruses have already been introduced into clinical practice. However, identification of the factors that underly the sensitivity of tumor species to oncolytic viruses or that modulate their clinical efficacy remains an important target. Here, we show that Coxsackievirus B5 (CVB5) demonstrates high oncolytic potential towards GBM primary cell species and cell lines. Moreover, 2-deoxyglucose (2DG), an inhibitor of glycolysis, potentiates the cytopathic effects of CVB5 in most of the cancer cell lines tested. The cells in which the inhibition of glycolysis enhanced oncolysis are characterized by high mitochondrial respiratory activity and glycolytic capacity, as determined by Seahorse analysis. Thus, 2-deoxyglucose and other analogs should be considered as adjuvants for oncolytic therapy of glioblastoma multiforme.

## 1. Introduction

Glioblastoma multiforme (GBM) represents a majority (48.6%) of malignant tumors of the central nervous system (CNS) [1] and accounts for more than 60% of all brain tumors in adults. Although this type of tumor can occur at any age, the peak of its incidence is between 55 and 60 years. GBM is considered one of the most malignant tumors, with an average median survival of 14–15 months after diagnosis for treated patients [2,3] and <4 months for those who do not receive treatment [4]. Current treatment options include surgical removal of the tumor, radiotherapy-based techniques, and chemotherapeutic agents [3]. However, their efficacy is very low. Up to 90% of patients who undergo surgical removal of the primary tumor or radiotherapy in a combination with standard therapy with temozolomide have relapsed close to the original site [5,6,7]. Radiotherapy has also significant side effects due to neuronal damage and radiation-induced necrosis [3]. Chemotherapy is based on various alkylating agents (temozolomide, lomustine, carmustine, procarbazine) or inhibitors of tubulin polymerization (i.e., vincristine) [8,9]. However, they often provide a very limited increase in the life span of a patient at a cost of severe side effects. Moderate efficacy is exhibited by neutralizing antibodies to growth factors such as VEGF (Bevacizumab). Therefore, the development of other strategies to combat glioblastoma is highly warranted.

Oncolytic viruses represent a rediscovered option to suppress the growth of GBM and other solid tumors [10,11,12,13,14]. Throughout the history of vaccine development and studying viral infection, spontaneous cancer regression and even remission upon viral infection were observed [15]. These initial observations paved the way for oncolytic viral therapy. The ability of oncolytic viruses to selectively destroy cancer cells has been mainly attributed to direct oncolysis (lysis of infected tumor cells) or to activation and enhancement of immune responses against tumor cells [10,11]. Notably, oncolytic viruses may restore the activity of the immune system, often suppressed by the tumor. Furthermore, oncolytic viruses can sensitize tumor cells to anticancer agents that induce apoptosis [16]. As a result, several oncolytic viruses have been introduced into clinical practice for the treatment of melanoma [17,18], head and neck cancer [19], and glioma [20].

The sensitivity of cells to viruses and the efficacy of oncolysis depend on many factors including the status of signaling pathways such as Ras/ERK, PI3K/Akt, and MAPK/ERK pathways [21,22], levels of expression of HIF-1α [23], and mutations in certain oncogenes (i.e., TP53 and PTEN) [24,25,26]. These factors as well as epigenetic changes ensure a favorable environment for virus replication exclusively in tumor cells [27]. Other factors determining the selectivity of a virus to the tumor cells include the altered architecture of the tumor tissue and increased vascularization [28].

Cytotoxic effects of oncolytic viruses can also be associated with the metabolic landscape of tumor cells (for example, [29]). Cancer cells have a rewired metabolism characterized by enhanced glycolysis that is uncoupled from the TCA cycle, thus leading to enhanced production and secretion of lactate. To fuel the TCA cycle and mitochondrial respiration, tumor cells exhibit metabolic plasticity [30]: they activate other pathways that produce AcCoA, i.e., by fatty acid oxidation [31] or from acetate [32] or other Krebs cycle intermediates such as a-ketoglutarate by glutaminolysis [33]. Such changes are driven by a variety of factors including mutations in oncogenes and tumor suppressors. For example, Gly12 substitution in K-Ras (Kirsten-rat sarcoma viral oncogene homolog), which is present in a wide spectrum of tumors including pancreatic, colorectal, and lung cancer [34], leads to enhanced glycolysis [35,36]. Glioblastomas exhibit activated EGF/Ras and PI3K signaling, mutations in the TERT promoter, or in isocitrate dehydrogenase 1 (IDH1), one of the key enzymes of the Krebs cycle [37]. PI3K/AKT/mTOR signaling reshapes all major metabolic pathways [38], TERT mutations were shown to promote the pentose phosphate pathway [39] and accumulation of glycogen [39], and mutated IDH1 produces the oncometabolite 2-hydroxyglutarate [40]. As a result, these characteristic metabolic traits can provide additional targets for the development of chemotherapeutic agents against this type of cancer [41].

The cytopathic effects of various viruses depend on the type of cell that is infected. As an example, SARS-CoV-2 is known to infect various cell lines including Vero E6, Calu3, Huh7, and Caco2, as well as cells overexpressing the ACE2 receptor (i.e., A549^ACE2^) (Vero, Calu, Caco, Huh7)] [42]. However, a cytopathic effect is visible only in the first two cell types [42]. Oncolytic Coxsackievirus type B3 (CVB3) was previously shown to induce cytopathic effects only in lung carcinoma with G12C K-Ras mutation and not to affect the viability of lung epithelial cells with wild-type K-Ras [43]. Therefore, it could be assumed that the oncolytic properties of viruses may depend on the metabolic status of their target cells.

The aim of our study was to investigate if pharmacological inhibitors of major metabolic pathways, i.e., glycolysis and mitochondrial respiration, can potentiate the cytopathic effect of oncolytic Coxsackievirus type B5 (CVB5) and other enteroviruses.

## 2. Materials and Methods

### 2.1. Materials

Metformin, phenformin, 2-deoxy-D-glucose (2DG), N-acetyl-L-cysteine (NAC), and galactose were from Sigma (Darmstadt, Germany). (3-(4,5-dimethylthiazol-2-yl)-2,5-diphenyltetrazolium bromide (MTT) was purchased from Dia m (Moscow, Russia). CellTracker^TM^ Green (CMFDA) dye and DAPI were from Thermo Fisher (Waltham, MA, USA). Glycolysis Stress Test Kit (Agilent, Santa Clara, CA, USA) and Mito Stress Test Kit (Agilent, USA) were used.

### 2.2. Cells

Primary cultures of GBM cells (GBM3821, GBM5522, GBM6067, and GBM6138) from surgically removed tumor tissues obtained from N.N. Burdenko Institute of Neurosurgery (Moscow, Russia) were described previously [44]. The HEK293T (CRL-3216), DBTRG-05MG (CRL-2020), A-172 (CRL-1620), HeLa cells (CCL-2), Vero (CCL-81), RD (CCL-136) lines were purchased from the ATCC collection. Human embryonic diploid fibroblasts (HEF) cells were a kind gift from the laboratory of Immunohistochemistry of the Serbsky National Scientific center. U251-MG cells were obtained from the collection of the laboratory of cell proliferation EIMB RAS. All stable cells were subjected to STR analysis using COrDIS Expert 26 kits on a Genetic analyzer 3500 (Waltham, MA, USA). The cultures were routinely checked for mycoplasma contamination using the MycoReport test (Evrogen, Moscow, Russia).

The cells were maintained in DMEM (Gibco, Jenks, OK, USA) supplemented with 10% FBS (HyClone, Cytiva, Sweden), 50 U/mL penicillin, and 50 µg/mL streptomycin at 37 °C in a humid atmosphere with 5% CO_2_. The cells were split 2–3 times a week when reaching sub-confluency by incubation with 0.25% trypsin-EDTA solution (PanEco, Moscow, Russia).

### 2.3. Viruses

The following virus strains were used: poliovirus vaccine strains (Sabin) of type 1, 2, and 3 (PV1, PV2, PV3, respectively); non-pathogenic oncolytic enteroviruses: Coxsackievirus B5—live enterovirus vaccine 14 (CVB5, GenBank: MG642820.1), Coxsackievirus A7—live enterovirus vaccine 8 (CVA7, GenBank: JQ041367.1), Coxsackie B6—live enterovirus vaccine 15 (CVB6, GenBank: JQ041368.1), Coxsackievirus A2 (CVA2), Coxsackievirus B3 (CVB3), Coxsackievirus A9 (CVA9) strains were obtained from the collection of the laboratory of cell proliferation. The viruses were propagated in HEK293T, Vero, or RD cell lines [44].

### 2.4. Cell Viability Assays

Cells were seeded on 96-well plates (TPP, Trasadingen, Switzerland) at a density of 5000 cells/well. Twenty-four hours later 2DG, metformin, or phenformin were added. After 24 or 48 h incubation, cell viability was assessed by MTT assay or using CellTracker reagent. In the MTT test, the medium was removed, and 100 µL of 5 mg/mL MTT solution in PBS was added to each well for 3 h followed by dissolving formazan crystals after medium aspiration in DMSO and absorbance measurement at 590 nm using ClarioStar^Plus^ microplate reader (BMG Labtech, Ortenberg, Germany). Alternatively, CellTracker dye was added to the media to the final concentration of 50 ng/mL, and after 10 min incubation at 37 °C, the cells were stained with DAPI (10 µg/mL concentration), and fluorescence was measured ClarioStar^Plus^ microplate reader, detecting CellTracker and DAPI emission wavelength, 517 and 470 nm, respectively. The ratio of life versus dead cells was calculated as the proportion of living cells (CellTracker) to totally stained cells (DAPI-stained cells).

Cytopathogenic effects of the viruses were measured using MTT and CellTracker dyes. The serial dilutions of viruses at the multiplicities of infection (MOI) 1000, 100, 10, 1, 0.1, 0.01, and 0.001 were added immediately after the addition of the compounds. As a control, the untreated cells were infected with the viruses at the same dilutions. Reed and Muench’s method was used for the quantification of the TCID50 value [45]. Three independent biological replicates were analyzed in quadruplicates.

### 2.5. Replication Assay

To determine the impact of 2DG on viral replication, cells were treated with 2DG and infected with viruses at MOI = 0.1, and the supernatants were harvested 48 h post-infection. Viral titers were assessed by infection of the HEK293T cell line with serial dilutions of the supernatants according to Reed and Muench [45]. Assessment of titration was performed 72 post-infection by MTT assay. Three independent biological replicates were analyzed in quadruplicates.

### 2.6. Metabolic Analysis

Glycolysis and mitochondrial respiration were assessed on an XFe24 Seahorse analyzer (Agilent Technologies, Santa-Clara, CA, USA) as described previously [46]. Briefly, the cells were seeded on XF24 Cell Culture Microplate 18 h before the analysis at a density of (4 × 10^4^ cells/well) in DMEM. For the MitoStress test, 30 min prior to analysis, the medium was changed to DMEM lacking phenol red and bicarbonate and supplemented with 25 mM glucose, 2 mM pyruvate, and 2 mM glutamine, and the plate was incubated at 37 °C in the absence of CO_2_. During the analysis ATP-synthase inhibitor oligomycin (1 µM), uncoupler FCCP (0.75 µM), and a mixture of complex I and III inhibitors rotenone and antimycin (1 µM each) were consequently added. Three readings were performed with 3 min intervals for each condition.

In the case of the GlycoStress test, 45 min before the analysis, the medium was changed to DMEM lacking phenol red dye, bicarbonate, and glucose and supplemented with 2 mM pyruvate and 2 mM glutamine. During the experiment, glucose, oligomycin, and 2DG were added to the final concentrations of 25 mM, 1 µM, and 50 mM, respectively.

The raw data were processed by Seahorse Wave Desktop software (Agilent Technologies).

### 2.7. Reverse Transcription and Real-Time PCR (RT-qPCR)

Total RNA was purified from cells grown on 6-well plates using a High Pure RNA Isolation Kit (Roche, Switzerland) according to the manufacturer’s instructions. cDNA was synthesized from 2 µg RNA using random hexamer primer and RevertAid reverse transcriptase during incubation at 42 for 1 h, with subsequent enzyme inactivation at 70 °C for 10 min and treatment with the recombinant DNAse I (Roche) during 15 min at 37 °C and similar inactivation.

Real-time PCR analysis was performed using the primers listed in Table S1 as described previously [47].

### 2.8. KRAS Mutation

KRAS mutation analysis was performed using KRAS Mutation Analysis Kit for Real-Time PCR (exons 2, 3, and 4) (EntroGen, Woodland Hills, CA, USA) in accordance with the manufacturer’s recommendations. Genomic DNA for the analysis was extracted from 10^6^ cells using the GeneJet Genomic DNA Purification kit (ThermoFisher Scientific, Waltham, MA, USA). Next RT-PCR was performed using LightCycler^®^ 96 system I (Roche Molecular Systems, Pleasanton, CA, USA).

### 2.9. Statistical Analysis

All experiments were performed in triplicates at least three times. All data are presented as means ± standard deviation (S.D.). Pairwise statistical significance was analyzed by a two-tailed t-test, whereas multiple comparisons were performed by ANOVA with the Tukey–Kremer post hoc test. In all cases a *p*-value ≤ 0.05 was considered statistically significant.

## 3. Results

### 3.1. Selection of Enterovirus with the Highest Oncolytic Activity towards GMB Cells

**Figure 1:**
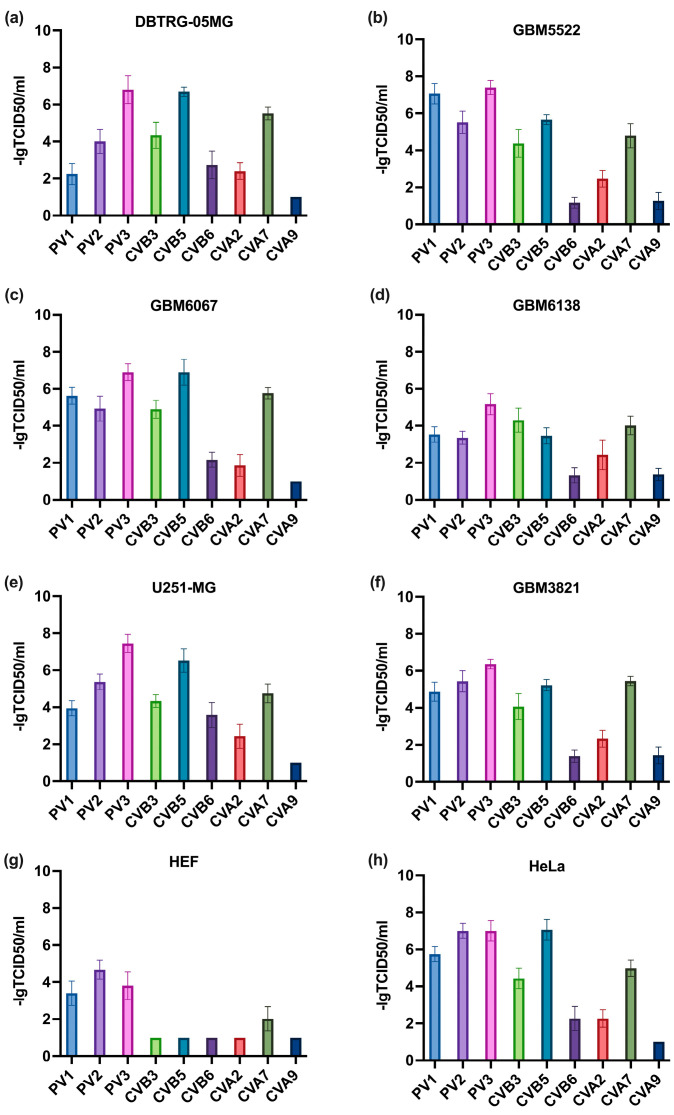
CVB5 exhibits the highest oncolytic activity towards glioblastoma multiforme (high grade) cell lines, stable glioblastoma cell lines: DBTRG-05MG (**a**), U251-MG (**e**), primary gliomas: GBM3821 (**f**), GBM5522 (**b**), GBM6067 (**c**), GBM6138 (**d**), and Hela cell line (**h**), as cancerous non-glioma cell line, and HEF (**g**) as non-cancerous cell line. The cells were seeded 24 h prior to infection and inoculated with MOIs from 0.001–1000 with polioviruses type 1–3 (PV1–3), Coxsackievirus A (CVA), or B (CVB). Cell viability was assessed 48 h later by MTT test. The depicted values are means ± S.D.

Our first goal was to select the enterovirus that demonstrates the highest oncolytic activity towards GBM cells. As such, four types of primary glioblastoma cells, described previously were used [44], alongside two standard GBM cell lines (U251 and DBTRG) and transformed human embryonal kidney cells HEK293T. Notably, all of these cells with the exception of U251-MG did not carry mutations in the K-RAS oncogene (not shown) that are associated with changes to metabolism [43]. In the U251-MG line, a Gly12Ser mutation was found. For negative control, we used human embryonal fibroblasts (HEF). As oncolytic viruses, polioviruses type I-III, and Coxsackievirus A (CVA) and B (CVB) were tested. Cells were infected with these viruses, and 48 h later oncolytic activity was assessed by MTT tests. The results, presented in Figure 1, clearly show that vaccine strains of polioviruses demonstrated cytotoxic activity towards the non-transformed fibroblasts (panel g). In contrast, CVB6, CVA2, and CVA9 exhibited rather low oncolytic effects towards GBM cells (Figure 1a–f). Since CBV5 showed higher activity than CVB3, it was used for further experiments.

### 3.2. Selection of Drug Concentrations

**Figure 2:**
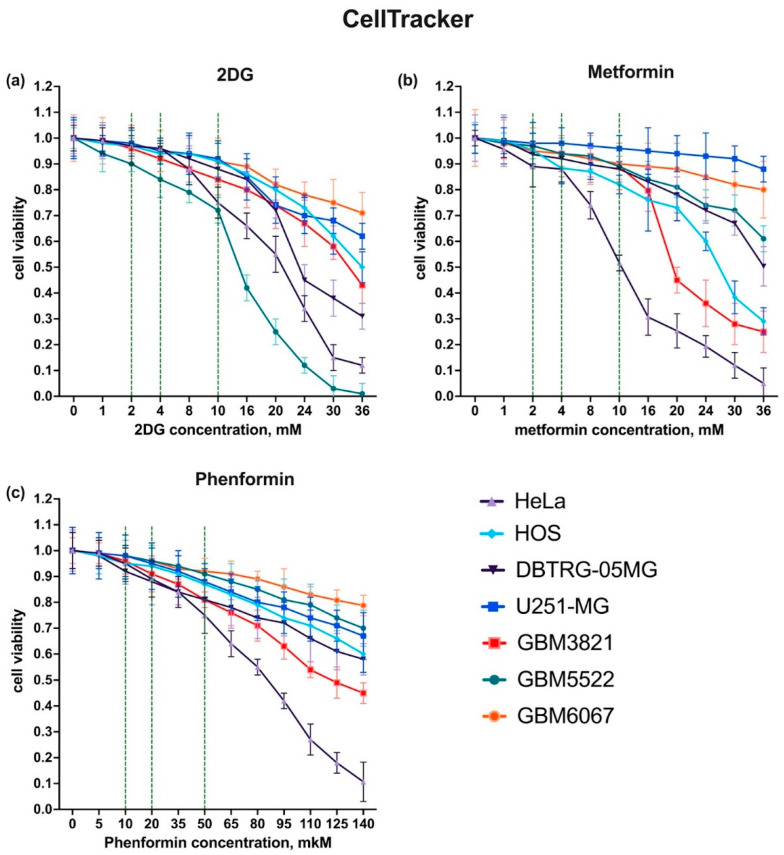
Cytotoxicity of metabolic inhibitors towards glioblastoma cell lines assessed using CellTracker reagent. Cells were seeded 24 h before exposure to increasing concentrations of 2DG (**a**), metformin (**b**), or phenformin (**c**). Cell viability was assessed 48 h after drug addition using CellTracker reagent.

As the aim of the study was to assess if inhibitors of metabolic pathways could enhance the potency of oncolytic enteroviruses against GBM, the next step was to choose the doses of compounds at which they do not affect cell growth or induce death. We used three compounds: 2DG (an inhibitor of glycolysis), metformin, and phenformin (inhibitors of mitochondrial respiratory complex I) [48]. Two standard cytotoxicity tests were used that were based on commonly used MTT or CellTracker reagents. The results for each cell line are shown in Figure 2 and Figure S1. For 2DG three concentrations were chosen: 2 and 4 mM, at which no significant changes to cell growth were observed, and 10 mM, where the compound moderately inhibited cell proliferation. Notably, in all these cases no cytotoxicity was observed, as shown by microscopy (Figures S2–S5). In the case of the GBM3821 line the drug affected cell proliferation, as in its presence, fewer cells were observed (Figure S3). The same concentrations were chosen for metformin, while phenformin was subsequently used at 10, 20, and 50 µM. Again, at the two lowest concentrations, the drugs did not compromise cell viability.

We should mention that in the MTT test, which is more widely used than the CellTracker reagent, significant effects of the drugs were noted (Figure S1). However, they did not correlate with cell number or viability as monitored by microscopy (Figure S2–S5). Therefore, this effect was probably associated with the dependency of MTT conversion into formazan products by oxidoreductases modulated by the metabolic status of a cell [49].

### 3.3. 2-Deoxyglucose Potentiates Cytopathic Activity of CBV5 towards Glioblastoma Cells

**Figure 3:**
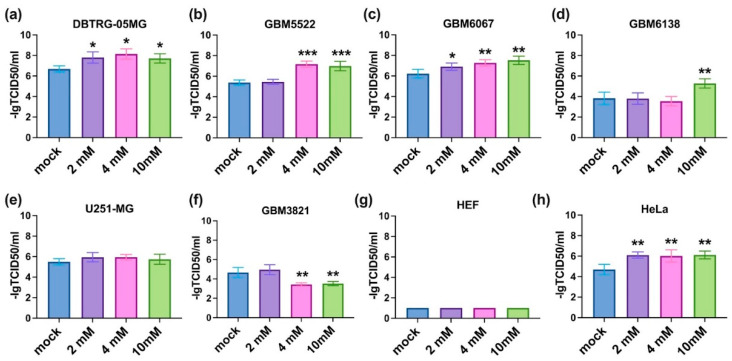
Oncolytic activity of CVB5 towards glioblastoma cells in the presence of 2DG increased in DBTRG-05MG (**a**), GBM5522 (**b**), GBM6067 (**c**), GBM6138 (**d**) and HeLa (**h**), decreased in GBM3821 (**f**) cell line, and did not change in U251-MG (**e**) and HEF (**g**) cell line. Cells were seeded 24 h before treatment with 2, 4, and 10 mM 2DG and simultaneous infection with CVB5 at MOIs of 0.001–1000. Viability was determined using CellTracker method 48 h later, * *p* ≤ 0.05, ** *p* ≤ 0.01, *** *p* ≤ 0.001.

**Figure 4:**
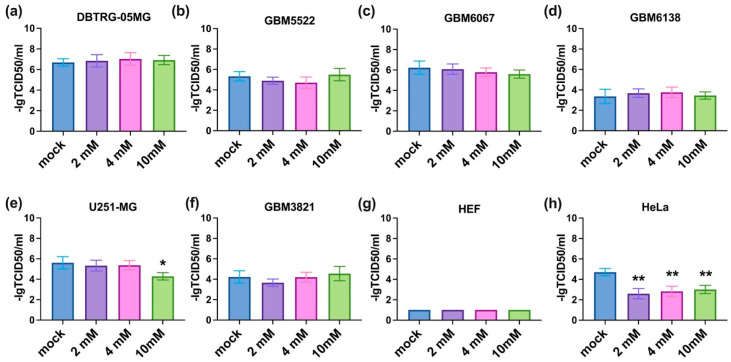
Oncolytic activity of CVB5 towards glioblastoma cells in the presence of metformin decreased in U251-MG (**e**) and HeLa (**h**) but not in DBTRG-05MG (**a**), GBM5522 (**b**), GBM6067 (**c**), GBM6138 (**d**), GBM3821 (**f**) and HEF (**g**) cell lines. Cells were seeded 24 h before treatment with 2, 4, and 10 mM metformin and simultaneous infection with CVB5 at MOIs of 0.001-1000. Viability was determined using CellTracker 48 h later, * *p* ≤ 0.05, ** *p* ≤ 0.01.

**Figure 5:**
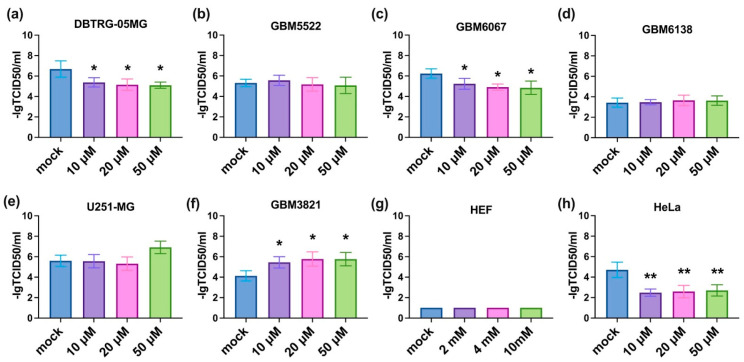
Oncolytic activity of CVB5 towards glioblastoma cells in the presence of phenformin decreased in DBTRG-05MG (**a**), GBM6067 (**c**), and HeLa (**h**), and increased in GBM3821 (**f**). In GBM5522 (**b**), GBM6138 (**d**), U251-MG (**e**), and HEF (**g**) cells it did not affect oncolytic activity of CVB5. Cells were seeded 24 h before treatment with 10, 20, and 50 µM phenformin and simultaneous infection with CVB5 at MOIs of 0.001–1000. Viability was determined using CellTracker method 48 h later, * *p* ≤ 0.05, ** *p* ≤ 0.01.

The next step was to investigate if the inhibitors of metabolic enzymes can potentiate the oncolytic activity of CVB5. All three compounds were added prior to infection in a standard titration assay. The results are summarized in Figure 3, Figure 4 and Figure 5. It can be seen that 2DG significantly augments the viral cytopathic effect in glioma DBTRG-05MG, GBM5522, and GBM6067 cell lines as well as in HeLa cells (Figure 3a–c,h). A similar effect, although at higher 2DG concentrations, was revealed in GBM6138 cells (Figure 3d). In contrast, in the GBM3821 cell line 2DG reduced the cytopathic effect of the virus (Figure 3f). Biguanides metformin and phenformin in general exhibited opposite effects: they inhibited the oncolytic activity of CVB5 in DBTRG-05MG, GBM6067, and HeLa cell lines (Figure 4h and Figure 5a,c,h) and potentiated it in GBM3821 cells (Figure 5f). Therefore, inhibition of glycolysis represents a promising strategy for the enhancement of the efficacy of oncolytic viruses.

### 3.4. Notably, 2DG Does Not Affect the Replication of CVB5

**Figure 6:**
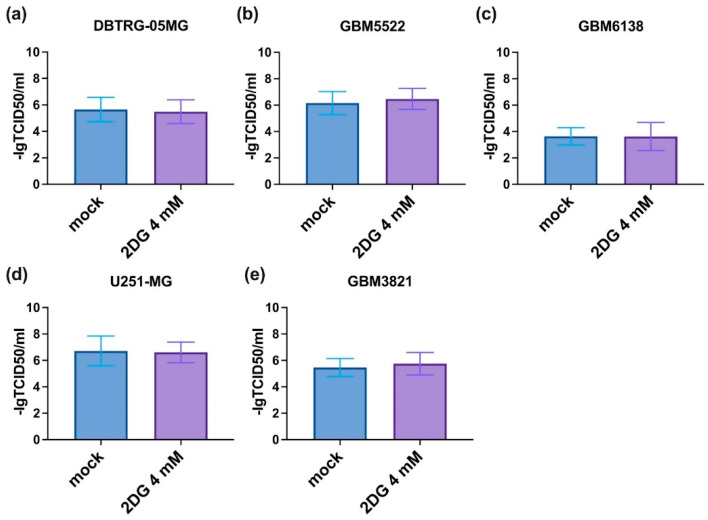
2DG does not affect CVB5 replication. HEK293T cells were infected with the conditioned medium from DBTRG-05MG (**a**), GBM5522 (**b**), GBM6138 (**c**), U251-MG (**d**) or GBM3821 (**e**) glioblastoma cells treated with 4 mM 2DG simultaneously with CVB5, harvested 48 h post-infection. Cell viability was assessed 72 h later using MTT assay. Values are means ± S.D.

One of the possible explanations for the enhanced cytopathic effect of CVB5 in the presence of 2DG could be an increased rate of viral replication. To test this assumption, we evaluated the production of infectious CVB5 particles in glioblastoma cell lines in the presence of the drug by harvesting supernatants and titrating them onto HEK293T cells according to standard procedures [50]. However, 2DG had no notable effect on the replication of the virus (Figure 6).

### 3.5. CBV5 Does Not Affect Cell Metabolism in Glioblastoma Cells

**Figure 7:**
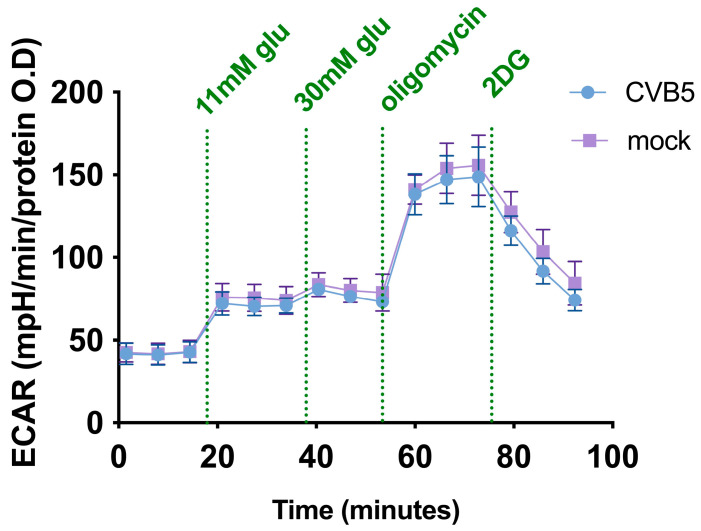
CVB5 does not affect the glycolytic activity of glioblastoma cells. DBTRG-05MG cells were infected with CBV5 at MOI 1, and the cells were subjected to GlycoStress assay according to manufacturer’s instructions at 7 h post-infection. In GlycoStress, glucose at final concentrations of 11 and 30 mM was followed by oligomycin (1 µM) and 2DG (50 µM) addition. Depicted values are means ± S.D.

Another possible mechanism of how subtoxic doses of 2DG could enhance oncolytic activity is via modulation of metabolism. To explore the impact of CVB5 on the metabolism of glioblastoma cells, the glycolytic status of the DBTRG-05MG cell line was analyzed by the Seahorse technology, which measures rates of extracellular acidification (ECAR) as a readout for anaerobic glycolysis [8]. After infection with CVB5 viral protein production and genome, replication was detected as early as 4 h post-infection (h.p.i.), and mature virions were released starting from 5–6 h.p.i., and the metabolism analysis was performed 7 h.p.i. to exclude any secondary effects of cell death. A typical ECAR curve is presented in Figure 7, while its analysis is given in the Supplement (Figure S6). It is clear that CBV5 does not affect the glycolytic status of glioma cells, at least during the early phase of infection.

### 3.6. Glioblastoma Cell Lines Sensitive to 2DG Exhibit Higher Levels of Glycolysis

**Figure 8:**
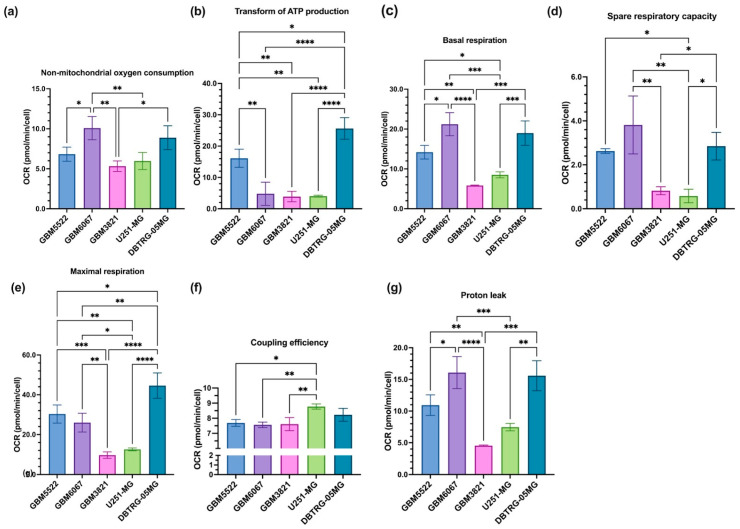
Enhanced oncolytic activity of CVB5 towards glioblastoma cell lines correlates with their basal mitochondrial respiration and spare respiratory capacity. Non-mitochondrial oxygen consumption (**a**), ATP production (**b**), basal (**c**) and maximum (**e**) respiration and spare respiratory capacity (**d**) as well as coupling efficiency (**f**) and proton leak (**g**) were assessed by the Seahorse technology in MitoStress assay. Oligomycin, (1 µM), FCCP (0.75 and 1.5 µM), and a mixture of antimycin and rotenone (1 µM each) were added. Values are means ± S.D. * *p* ≤ 0.05, ** *p* ≤ 0.01, *** *p* ≤ 0.001, **** *p* ≤ 0.0001.

**Figure 9:**
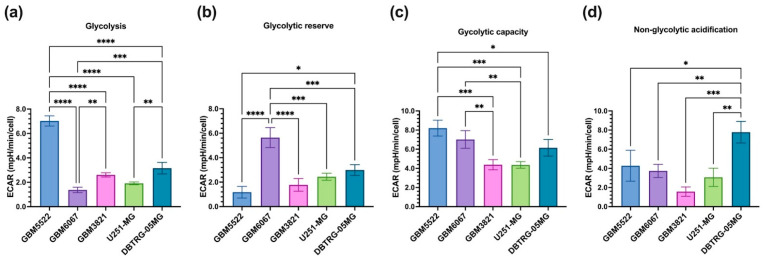
Enhanced oncolytic activity of CVB5 towards glioblastoma cell lines correlates with their glycolytic capacity. Basal glycolysis (**a**), glycolytic reserve (**b**) and maximum capacity (**c**) as well as non-glycolytic acidification (**d**) were assessed by the Seahorse technology in GlycoStress assay. Glucose was added to the final concentrations of 11 and 30 mM followed by addition of oligomycin (Oligo, 1 µM), and 2DG (50 µM). Values are means ± S.D, * *p* ≤ 0.05, ** *p* ≤ 0.01, *** *p* ≤ 0.001, **** *p* ≤ 0.0001.

To identify the features of glioblastoma cell lines that mediate the increase in the oncolytic effects by 2DG, the metabolic activity of three 2DG-sensitive and two resistant cell lines was assessed by Seahorse technology in MitoStress (Figure 8 and Figure S7a) and GlycoStress (Figure 9 and Figure S7b) tests. In the MitoStress assay, two concentrations of uncoupler (FCCP) were taken to exclude possible different sensitivity of the cell lines to this agent. Similarly, in the GlycoStress test glycolysis efficiency was analyzed using two different concentrations of glucose. The “2DG-sensitive” cell lines DBTRG-05MG, GBM5522, and GBM6067 exhibited much higher basal and maximum respiratory activity as well as spare respiratory capacity and proton leak (Figure 8c–e,g), while no correlation with ATP production was noted. We also did not find any correlation with basal glycolytic activity (Figure 9a) or glycolytic reserve (Figure 9b). However, the cells, in which 2DG potentiated oncolytic activity, had higher glycolytic reserves (Figure 9c). To sum up this section, “sensitivity” to 2DG is associated with increased respiration and maximum glycolytic capacity.

### 3.7. The Effect of 2DG Is Not Redox-Sensitive

**Figure 10:**
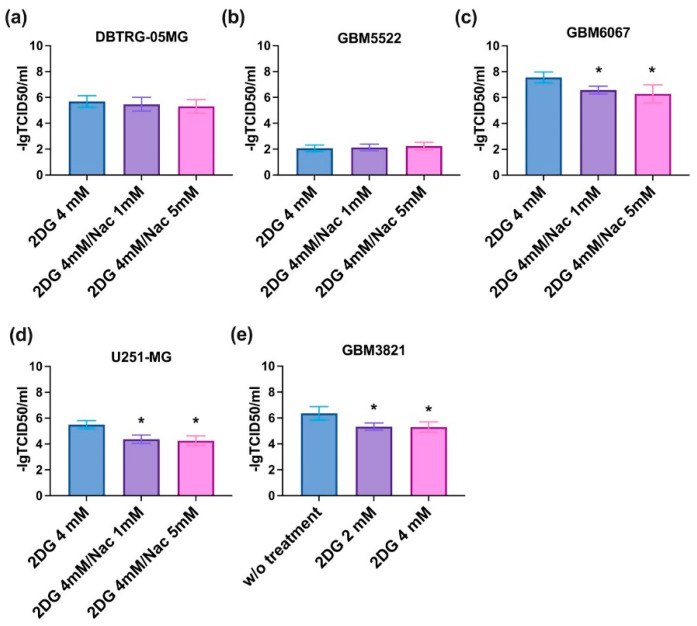
Effect of antioxidant N-acetylcysteine (NAC) on cytopathic effect of CVB5 in the presence of 2DG. DBTRG-05MG (**a**), GBM5522 (**b**), GBM6067 (**c**), U251-MG (**d**) or GBM3821 (**e**) cells were seeded day before treatment with 4 mM 2DG, NAC (1 or 5 mM), and simultaneous infection with CVB5 at MOIs of 0.001-1000. Viability was assessed 48 h post-infection. Values are means ± S.D. * *p* < 0.05.

Various cellular metabolic pathways including glycolysis are tightly linked to the production of reactive oxygen species (ROS). To unveil if ROS play a role in the synergistic effect of CVB5 2DG, the effect of the antioxidant N-acetylcysteine (NAC) was evaluated. In the 2DG-sensitive cell lines, DBTRG-05GM and GBM5522 NAC did not affect the oncolytic activity of the virus. However, in the 2DG-sensitive GBV6067 cells and the two 2DG-insensitive cell lines, U251-MG and GBM3821 it caused a statistically significant inhibition of the cytopathic effects (Figure 10).

### 3.8. Notably, 2DG Increases Cytopathic Effect towards Glioblastoma Cells of a Wide Array of Enteroviruses

**Figure 11:**
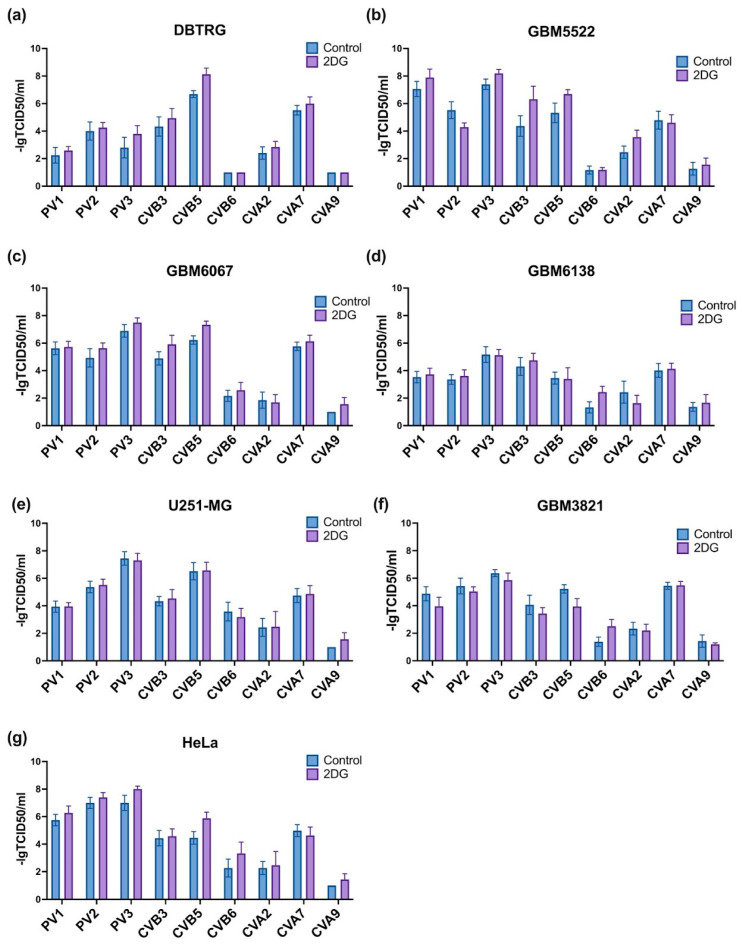
Influence of 2DG on the cytopathic effects of polioviruses and Coxsackieviruses depends on the virus as well as the cell line. DBTRG (**a**), GBM5522 (**b**), GBM6067 (**c**), GBM6138 (**d**), U251-MG (**e**), GBM3821 (**f**) or HeLa (**g**) cells were seeded day before treatment with 4 mM 2DG and simultaneous infection with viruses at MOIs of 0.001–1000. Viability was assessed after 48 h using MTT assay. Values are means ± S.D.

To evaluate if 2DG can enhance the oncolytic activity of additional RNA viruses, we tested poliovirus types 1-3 as well as Coxsackieviruses A and B. The results are presented on Figure 11. We found that in DBTRG-05MG as well as in HeLa cells 2DG enhances the activity of CVB5 only, whereas in two other “sensitive” cell lines it augmented cell death in the case of CBV3 as well (Figure 11a–c,g). In some cases, 2DG also increased the cytopathic effect of CVB6 or CVA2, but in neither case, the synergism reached the levels observed between 2DG and CBV5. Therefore, 2DG can be regarded as an agent that enhances the oncolytic activity of CBV5 and CBV3.

### 3.9. RT-qPCR

**Figure 12:**
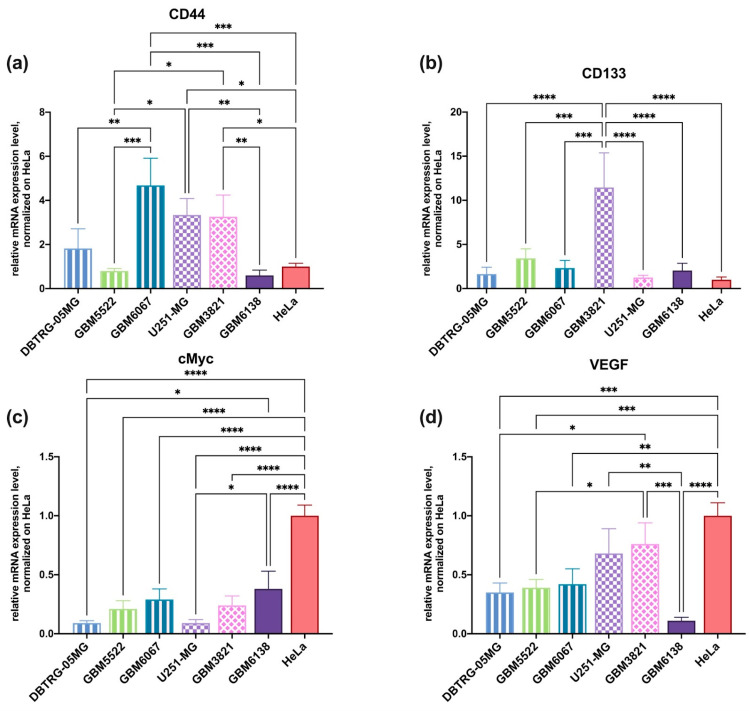
Levels of transcription of genes that are the markers of cancer stem cells. The cells were cultivated in standard condition, and CD44 (**a**), CD133 (**b**), cMyc (**c**) and VEGF (**d**) mRNA levels were assessed by RT-qPCR, GUS mRNA levels were used for normalization. The data were analyzed using ∆∆Ct methods, normalized to HeLa cells line. Values are means ± S.D. * *p* ≤ 0.05, ** *p* ≤ 0.01, *** *p* ≤ 0.001, **** *p* ≤ 0.0001.

We finally asked whether cellular sensitivity to the cytopathic effects of oncolytic viruses and 2DG is associated with differentiation status and features of cancer stem cells (also referred to as tumor-initiating cells) (CSC). CSCs are known to have the highest capacity to drive tumorigenesis [51]. They also often confer resistance to anticancer agents [52]. Among the markers of glioma CSCs are CD44, CD133, and cMyc [53,54] and increased levels of VEGF expression [55]. Therefore, the transcript levels of these markers were quantified on the above-used cell lines. As shown in Figure 12, the sensitivity of the enhancement of the cytopathic effect of CBV5 by 2DG did not correlate with the expression of any of these genes. Therefore, one can assume that 2DG can be used in combination with CVB5 irrespectively to the differentiation status of glioma.

## 4. Discussion

In this study, we showed that the Coxsackievirus type 5 exhibits strong oncolytic activity towards high-grade glioblastoma multiforme cells, and in most cases, this activity can be augmented with 2-deoxyglucose, an inhibitor of glycolysis. The stimulation occurs in cells with elevated glycolytic and respiratory phenotypes.

Oncolytic viruses are regarded as a promising tool for the treatment of a variety of tumors [10,12,13,14,56]. Among various types of cancer, GBM represents one of the most challenging targets, as patients with this type of brain tumors have poor prognosis due to low efficacies of treatment, very high rates of relapse, and severe side effects of the treatment [3,4,6,7]. To date, oncolytic activity towards GBM cells has been shown by wild-type and genetically engineered adenoviruses [57], Newcastle disease virus [58], Zika [59,60] and Chukingunya viruses [61], poxviruses [62], and HSV-1 [63], with the latter being introduced to clinical practice [64,65]. Cytopathic effects against GBM are also exhibited by vesicular stomatitis virus (VSV) carrying Lassa [66] or Ebola [67] virus proteins. Our group previously expanded this list by showing the oncolytic activity of coxsackie and polioviruses towards glioma cells [44]. In the current study, we presented a detailed analysis of the sensitivity of GMB cell lines to various strains of these viruses.

Inhibitors of metabolic enzymes are often considered anticancer agents alone or in combination with standard therapy, with several of them successfully used in clinical practice [68]. Although they often do not exhibit sufficient efficacy alone, they can increase the potency of standard therapy [63,69] and/or help to overcome the resistance of tumor cells to low molecular weight anticancer drugs and immunotherapy [70,71]. One of the most pronounced examples is Eflornithine, an inhibitor of polyamine biosynthesis, which shows the outstanding capacity to both reduce rates of relapse of neuroblastoma and to overcome the resistance of relapsed tumors to a standard combination of drugs [72,73,74]. All this underlines the potential of inhibitors of metabolic enzymes as promising anticancer agents.

Like many other types of cancer, glioblastoma multiforme is characterized by a significantly rewired metabolism. GBM exhibits markedly elevated glycolysis (as well as mitochondrial respiration) [75] which is at least partially due to enhanced RAS signaling [76] and amplification of MYC and MYCN genes [77]. In addition, the tumor is highly dependent on de novo fatty acid and lipid biosynthesis [31]. Acetyl-coenzyme A synthetase (ACSS2) is one of the key enzymes in GBM metabolism, as it converts acetate into AcCoA that not only serves as a precursor for fatty acid biosynthesis but also feeds TCA cycle together with glucose [32]. However, at hypoglycemic conditions, the TCA cycle can rely on the production of fatty acid and ketone catabolism. Nevertheless, inhibition of glycolytic enzymes is considered a promising strategy for both treatment of brain tumors and their sensitizing to chemo- and radiotherapy.

Here, we evaluated 2DG, a glycolysis inhibitor, as well as two inhibitors of the mitochondrial respiration system (metformin and phenformin), as possible agents to enhance the oncolytic activity of enteroviruses towards GBM. We show that 2DG increased the oncolytic activity of CVB5 towards three out of five primary GBM cultures and one out of two classical glioma cell lines. In the case of the fourth primary cell culture (GBM6138) similar increase was also observed, albeit at higher concentrations of the drug. In contrast, metformin and phenformin generally caused opposite effects on the oncolytic activity of CVB5. Similar results were recently shown by the Al-Shammari group for the Newcastle disease virus acting as an oncolytic towards breast cancer cells [78,79]. This group triggered glucose deprivation not only with 2DG [78] but also with acarbose which is an inhibitor of alpha-glucosidase and amylase [79]. Another example is the use of dichloroacetate which promotes a shift from anaerobic glycolysis to oxidative phosphorylation for the enhancement of oncolysis by the measles virus towards GBM [80].

Search for biomarkers for personalized virotherapy remains one of the most challenging tasks in the field [81]. In our case, since 2DG enhances the oncolytic activity of Coxsackievirus in a majority but not all GBM species, the key goal is a pre-selection of patients for such therapy. To find biomarkers, we analyzed the presence of mutations at the Gly12 position of K-Ras and evaluated the metabolic phenotype of cells with variable “sensitivity” to 2DG. All the lines except for U251-MG had wild-type K-RAS (data not shown). At the same time, it was found that an increase in CVB5 potency by the drug occurs for the GBM cells with high respiratory phenotype as well as with elevated glycolytic capacity. Therefore, pre-evaluation of the rate of oxidative phosphorylation in tumor samples may be used in the future to identify patients with gliomas who will respond to a combination of CVB5 and 2DG.

2-Deoxyglucose can be considered a safe drug. To date, it has been evaluated as an experimental anti-SARS-COV-2 drug. As reported in a single paper [82], this compound at a daily dose of 90 mg/kg in a combination with the standard treatment provided clinical benefits for COVID-19 patients such as shortening time to achieve normal oxygen saturation levels and as a result time to discharge from hospital. The treatment was rather safe, as only 30% of patients reported side effects, which in most cases were mild. Similarly, in a phase I/II trial (NCT00633087), 30 mg/kg of 2DG was administered for 2–3 weeks to patients with advanced prostate cancer. The most common adverse effects were prolonged QTc interval (33%) and fatigue (42%). 2DG has also been used to increase the tolerance of radiotherapy against glioma, again, with good clinical effects [83]. Therefore, this drug as well as its analogs can be regarded as promising agents to increase the efficacy of virotherapy.

One of the challenges in treating cancer and achieving sustained response is the eradication of tumor-initiating cells, also referred to as cancer stem cells (CSCs) [51]. These cells generally comprise a small fraction of tumors, but they show lower sensitivity to anticancer drugs. Thus, initial responses of the tumor to treatment may reflect the sensitivity of differentiated cells, while the following relapse is due to the initiation of CSC growth followed by reconstitution/replenishment of the tumor volume [52,84]. Notably, the metabolic landscape and redox status of CSC differ from those of differentiated tumor cells. While standard cancer cells are mostly dependent on anaerobic glycolysis, CSC generally relies on oxidative phosphorylation [84]. Therefore, metformin and other inhibitors of respiratory complexes are considered tools to shift metabolism to glycolysis and thus increase the sensitivity to other agents [48]. In our study, we did not assess the activity of enteroviruses and metabolic inhibitors specifically towards CSCs but assessed if the effect of 2DG was associated with the expression of several markers of CSCs. We failed to find any correlation between their expression and the impact of the inhibitor of glycolysis. Therefore, 2DG is likely to act on both differentiated GBM cells and glioma CSCs.

## 5. Conclusions

Thus, we showed that the inhibitors of glycolysis may significantly enhance the cytopathic effects of oncolytic enteroviruses including Coxsackievirus type B5 as well as B3. This effect occurs in cells with high mitochondrial respiratory activity and glycolytic capacity. Although this effect yet has to be verified in in vivo models, it may open a new direction to the treatment of glioblastoma and possibly other types of non-curable human tumors.

## Data Availability

Not applicable.

## Supplementary Materials

The following supporting information can be downloaded at: https://www.mdpi.com/article/10.3390/cancers14225611/s1, Table S1. The list of primers used for gene expression analysis by quantitative real-time PCR; Figure S1. Cytotoxicity of metabolic inhibitors towards glioblastoma cell lines assessed using MTT reagent; Figure S2. Morphology of DBTRG-05MG cells treated with 2-deoxyglucose for 48 h; Figure S3. Morphology of GBM3821 cells treated with 2-deoxyglucose for 48 h; Figure S4. Morphology of GBM5222 cells treated with 2-deoxyglucose for 48 h; Figure S5. Morphology of GBM6067 cells treated with 2-deoxyglucose for 48 h; Figure S6. CVB5 does not affect the glycolytic activity of glioblastoma cells; Figure S7. Enhanced oncolytic activity of CVB5 towards glioblastoma cell lines correlates with their basal mitochondrial respiration and spare respiratory capacity and glycolytic reserve.
