# Supplementary material for: 2-Deoxyglucose, an Inhibitor of Glycolysis, Enhances the Oncolytic Effect of Coxsackievirus
Source: Cancers (Basel). 2022 Nov 15;14(22):5611. doi: 10.3390/cancers14225611 (PMC9688421; doi:10.3390/cancers14225611)
Supplement: Supplementary file 1 [file cancers-14-05611-s001.zip › cancers-2024966-supplementary.pdf]

Supplementary data

2-Deoxyglucose, an inhibitor of glycolysis, enhances the oncolytic effect of Coxsackievirus

Pavel O. Vorobyev [1], Dmitry V. Kochetkov [1], Peter M. Chumakov [1], Natalia F. Zakirova [1], Sofia I. Nefedorova [1], Konstantin V. Vasilenko [1,2], Olga N. Alekseeva [1], Sergey N. Kochetkov [1], Birke Bartosch [3], Anastasiya V. Lipatova [1,*] and Alexander V. Ivanov [2,*]

1 Center for Precision Genome Editing and Genetic Technologies for Biomedicine, Engelhardt institute of molecular biology, Russian academy of sciences
2 Pirogov Russian National. Medical University
3 Lyon Cancer Research Center / INSERM U1052
* Correspondence: lipatovaanv@gmail.com (A.L.) or aivanov@yandex.ru (A.I.)

Table S1. The list of primers used for gene expression analysis by quantitative Real Time PCR

Title 1	Orientation	Sequence (5′→3′)
cMyc	sense	GGCTCCTGGCAAAAGGTCA
	antisense	CTGCGTAGTTGTGCTGATGT
CD133	sense	ATGGCCCTCGTACTCGGC
	antisense	TCAATGTTGTGATGGGCTTGT
CD44	sense	ATGGACAAGTTTTGGTGGCA
	antisense	TTACACCCCAATCTTCATGTC
VEGF	sense	CTTGCCTTGCTGCTCTAC
	antisense	TGGCTTGAAGATGTACTCG
GUS	sense	CGTGGTTGGAGAGCTCATTTGGAA
	antisense	ATTCCCCAGCACTCTCGTCGGT

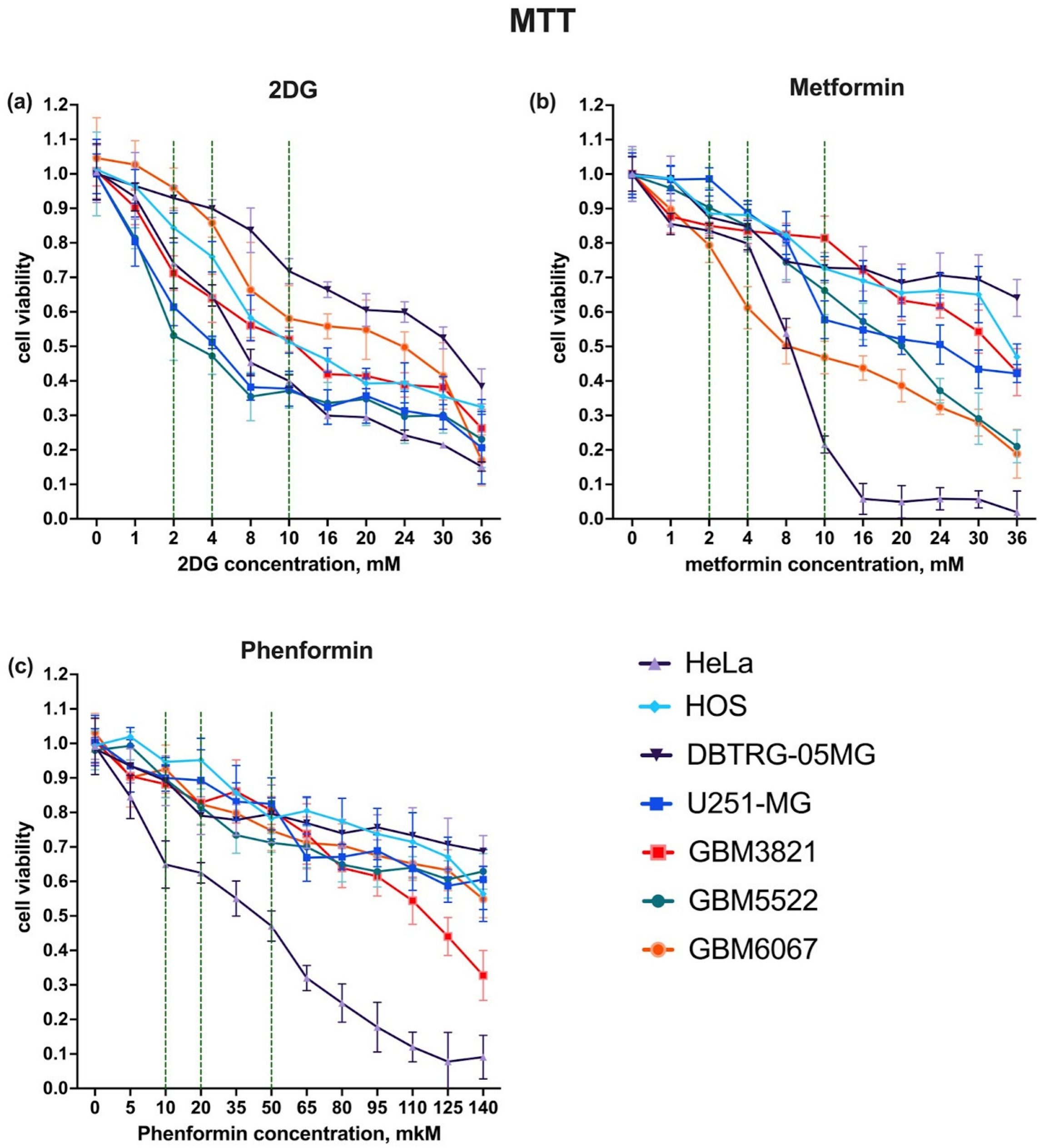

Figure S1. Cytotoxicity of metabolic inhibitors towards glioblastoma cell lines assessed using MTT reagent. Cells were seeded 24 hours before exposure to increasing concentrations of 2-DG (**a**), metformin (**b**) or phenformin (**c**). Cell viability was assessed 48 h after drug addition using MTT reagent.

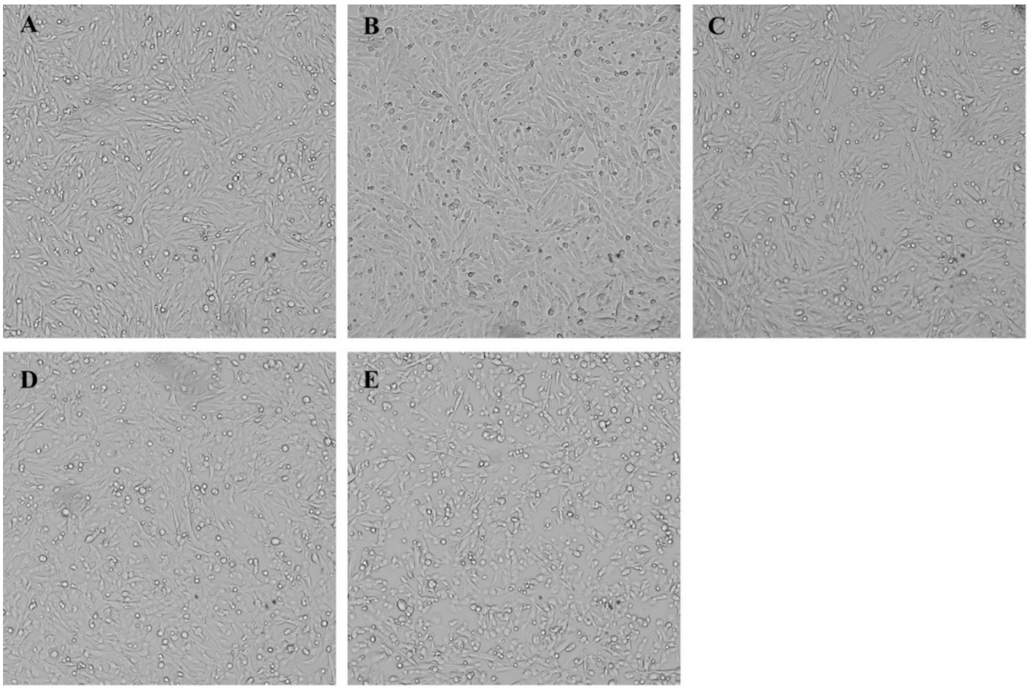

Figure S2. Morphology of DBTRG-05MG cells treated with 2-deoxyglucose for 48 h. (A) Untreated, (B-E) – treated with increased concentration of 2, 4, 10 or 36 mM 2DG. Cells were seeded 24 hours before treatment with mentioned above concentration of 2DG. The images were obtained by phase-contrast inverted microscopy at 10x magnification.

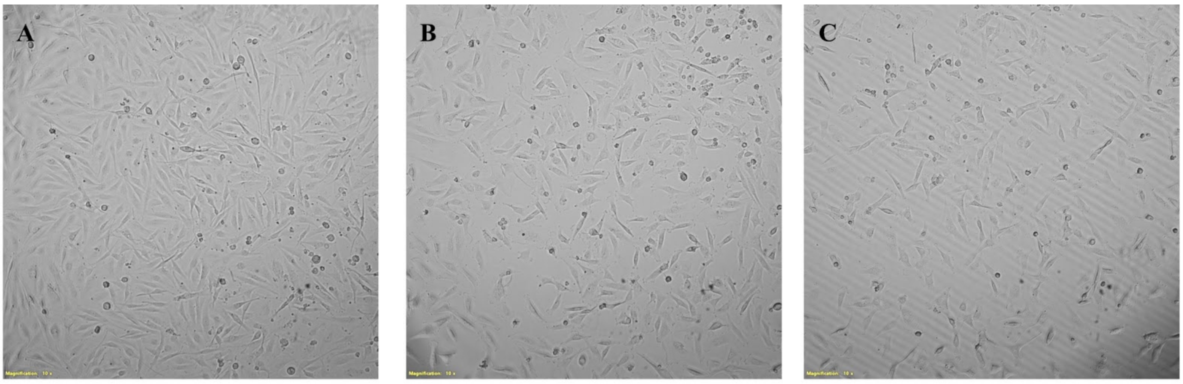

Figure S3. Morphology of GBM3821 cells treated with 2-deoxyglucose for 48 h. (A) Untreated, (B-C) – treated with 2 or 4mM 2DG. Cells were seeded 24 hours before treatment with mentioned above concentration of 2DG. The images were obtained by phase-contrast inverted microscopy at 10x magnification.

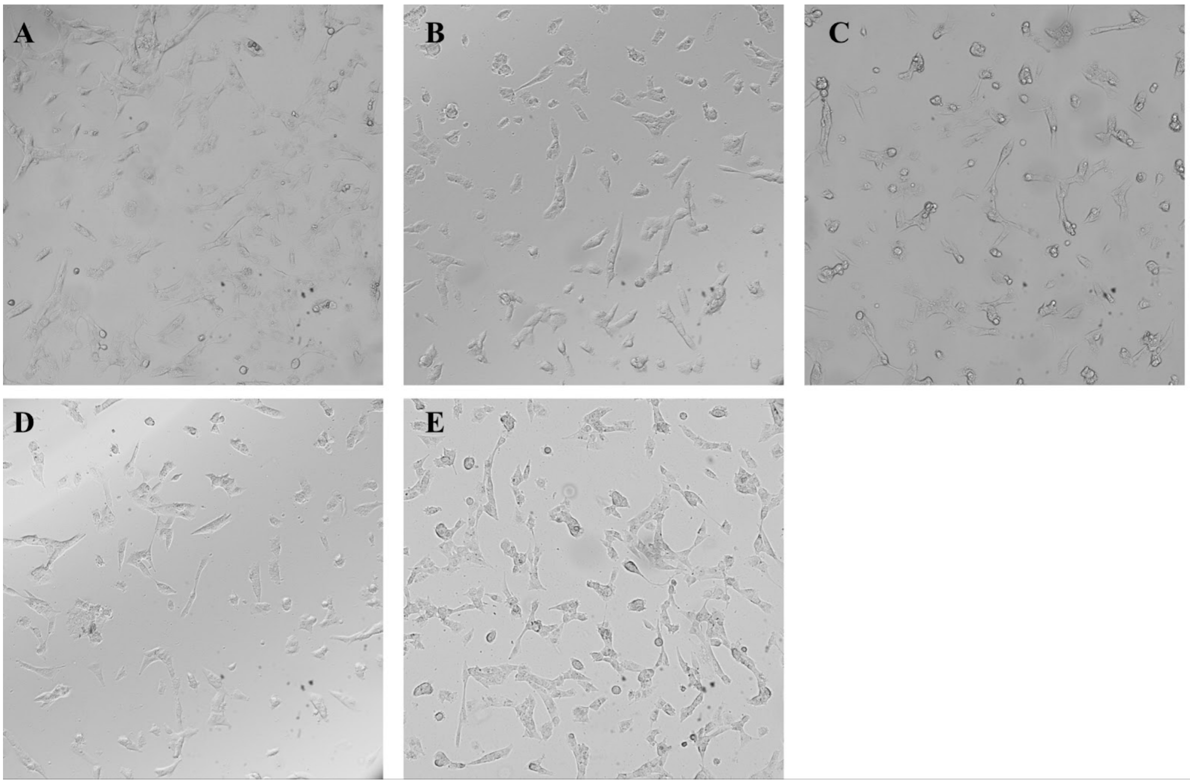

Figure S4. Morphology of GBM5222 cells treated with 2-deoxyglucose for 48 h. (A) Untreated, (B-E) – treated with 2, 4, 10 or 36 mM 2DG. Cells were seeded 24 hours before treatment with mentioned above concentration of 2DG. The images were obtained by phase-contrast inverted microscopy at 10x magnification.

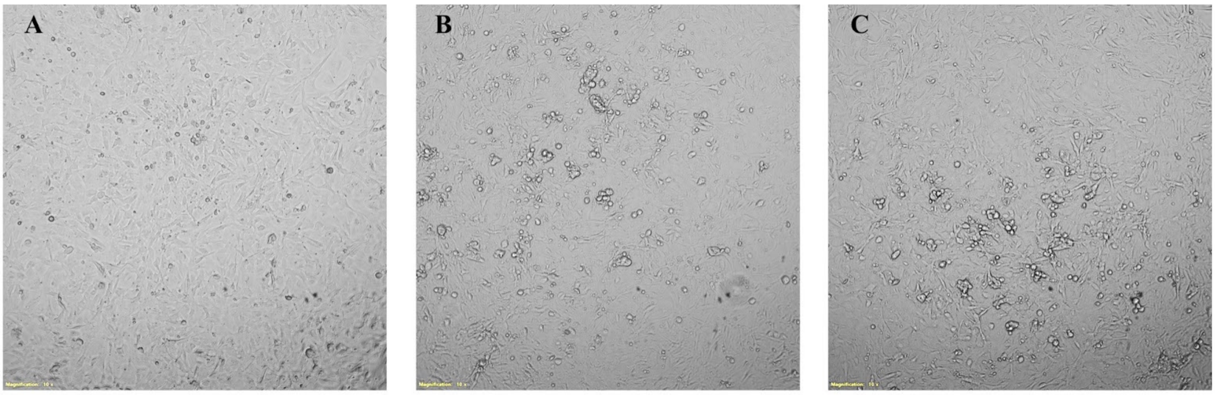

Figure S5. Morphology of GBM6067 cells treated with 2-deoxyglucose for 48 h. (A) Untreated, (B-C) – treated with 2 or 4mM 2DG. Cells were seeded 24 hours before treatment with mentioned above concentration of 2DG. The images were obtained by phase-contrast inverted microscopy at 10x magnification.

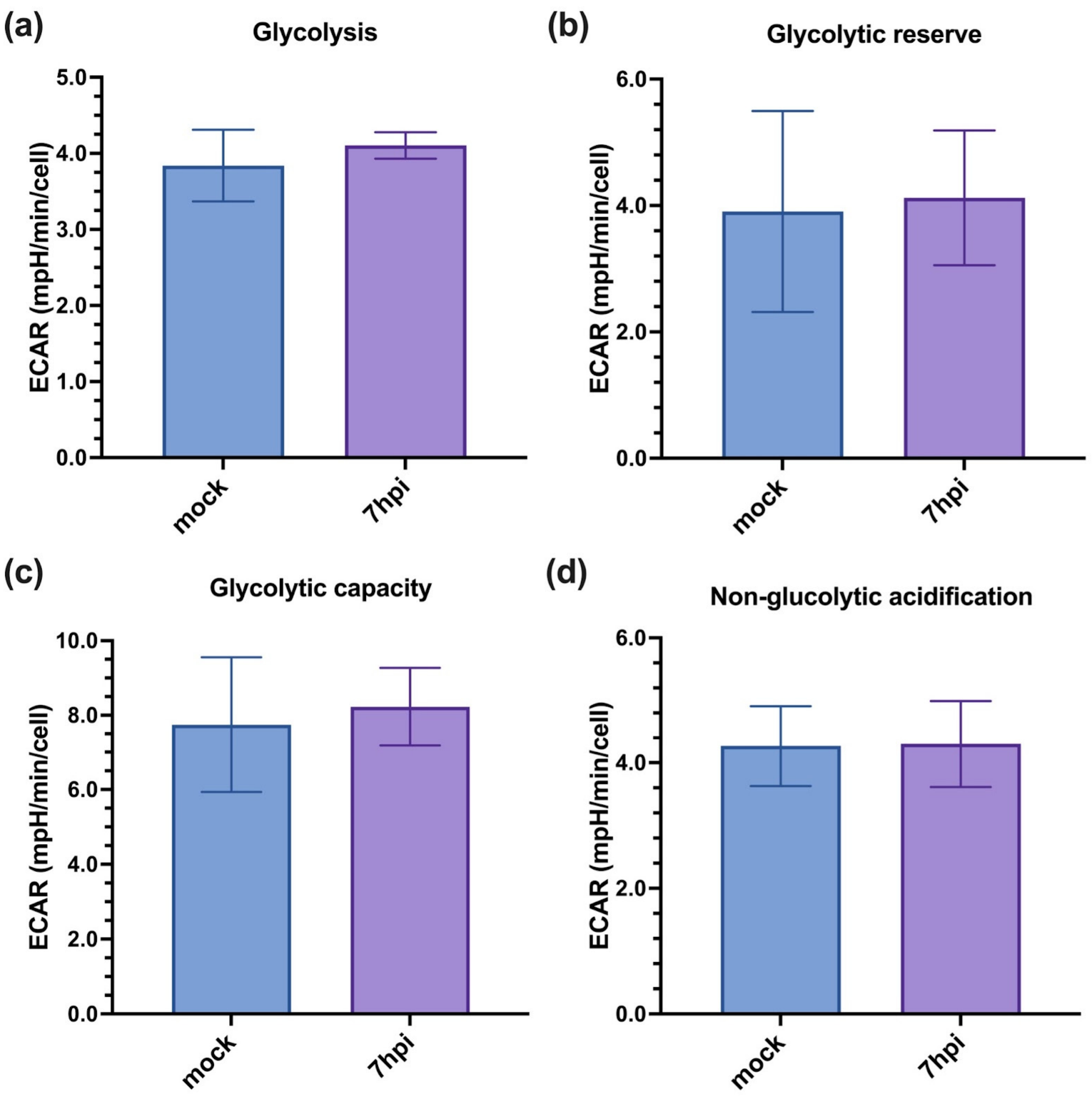

Figure S6. CVB5 does not affect the glycolytic activity of glioblastoma cells. DBTRG-05MG cells were infected with CBV5 at MOI 1, and the cells were subjected to GlycoStress assay according to manufacturer's instruction at 7 h post-infection. In GlycoStress, glucose at final concentrations of 11 and 30 mM was followed by oligomycin (1 μM) and 2-DG (50 μM) addition. Depicted values are means ±S.D.

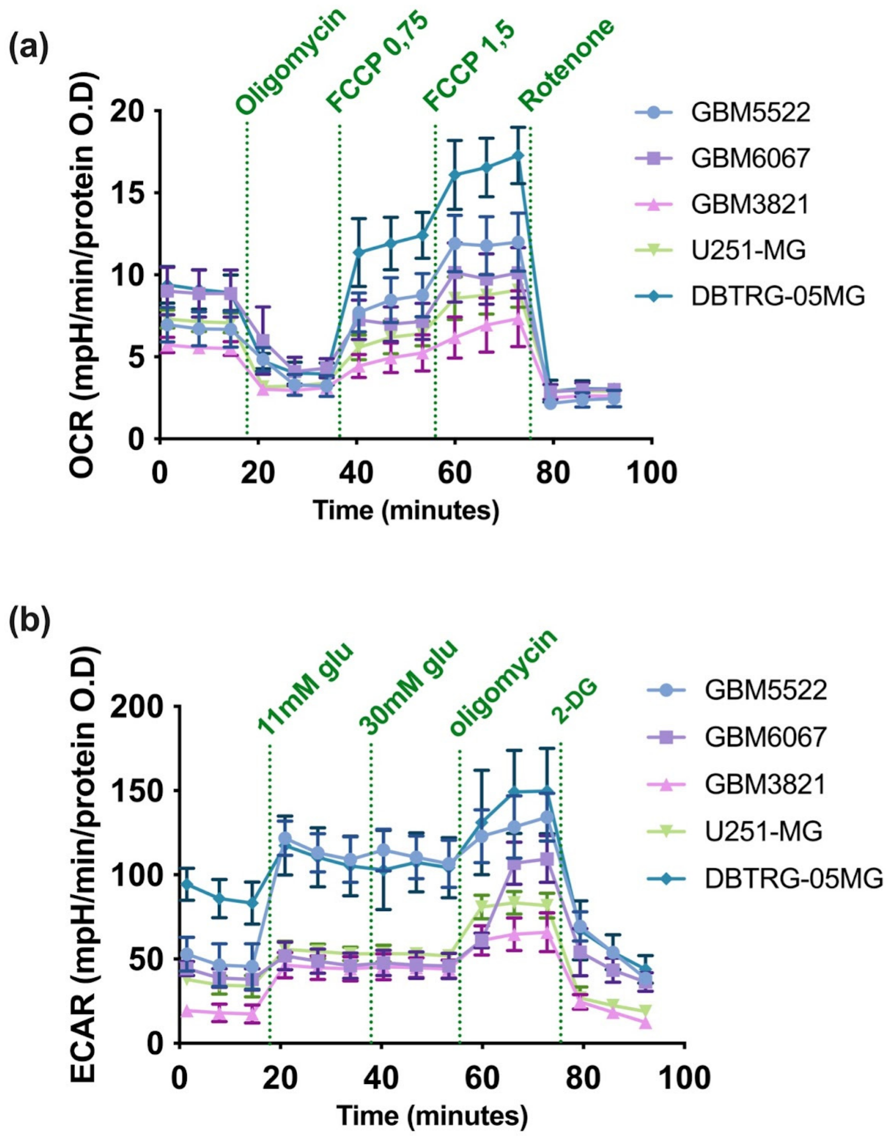

Figure S7. Enhanced oncolytic activity of CVB5 towards glioblastoma cell lines correlates with their basal mitochondrial respiration and spare respiratory capacity and glycolytic reserve. Parameters of mitochondrial respiration were assessed by the Seahorse technology in MitoStress assay. Oligomycin, (1 μM), FCCP (0.75 and 1.5 μM), and a mixture of antimycin and rotenone (1 μM each) were added. In GlycoStress glycose was added to the final concentration of 11 and 30 mM followed by addition of oligomycin (Oligo, 1 μM), and 2-deoxyglucose (50 μM). Values are means ±S.D.
